# Understanding the development of neural abnormalities in adolescents with mental health problems: A longitudinal study

**DOI:** 10.1016/j.nicl.2025.103885

**Published:** 2025-09-23

**Authors:** Jiangyun Hou, Laurens van de Mortel, Weijian Liu, Shu Liu, Arne Popma, Dirk J.A. Smit, Guido van Wingen

**Affiliations:** a Amsterdam UMC Location University of Amsterdam, Department of Psychiatry, Meibergdreef 5, Amsterdam, the Netherlands; b Amsterdam Neuroscience, Amsterdam, the Netherlands; c Amsterdam Public Health, Amsterdam, the Netherlands; d Danish Research Centre for Magnetic Resonance, Department of Radiology and Nuclear Medicine, Copenhagen University Hospital Amager and Hvidovre, Hvidovre, Denmark; e Key Laboratory of Genetic Evolution & Animal Models, National Research Facility for Phenotypic & Genetic Analysis of Model Animals (Primate Facility), National Resource Center for Non-Human Primates, Kunming Institute of Zoology, Chinese Academy of Sciences, Kunming, China

**Keywords:** Mental health problems, Adolescent, Neuro abnormalities, Neurodevelopment, Multi-modalities

## Abstract

**Background:**

Many mental health problems are neurodevelopmental in nature and have an onset during childhood. These disorders are associated with neural abnormalities, but it is unclear when these emerge and how this relates to the development of different mental health problems.

**Methods:**

We identified children who developed mental health problems over two years and controls who remained healthy from the Adolescent Brain Cognitive Development (ABCD) study. Six mental health conditions (N = 58 to N = 173) were compared to controls (N = 2500) using separate linear models to assess group differences at baseline and in neurodevelopment for six disorders and six modalities. Shared neurodevelopmental changes were assessed by comparing spatial patterns of brain alterations across conditions at baseline and over time.

**Results:**

The baseline data showed brain-wide abnormalities in children who later developed mental health problems, which were comparable between internalizing problems and different from externalizing problems. Brain-region specific abnormalities were limited to individuals who later developed oppositional defiant symptoms. The longitudinal data showed differential neurodevelopmental trajectories for specific brain regions in adolescents who developed ADHD, conduct and anxiety problems, as well as brain-wide neurodevelopmental abnormalities that were comparable between mental health problem groups compared to controls.

**Conclusions:**

Our findings reveal both shared and problem-specific neural abnormalities, providing critical insights into the evolving neurobiological mechanisms that underlie both shared and distinct mental health problems, highlighting how disorder-specific and transdiagnostic brain abnormalities emerge across development.

## Highlights

• Neural abnormalities mostly emerge with onset of adolescent mental health problems.
• Conduct and oppositional defiant symptoms show premorbid brain function differences.
• Brain developmental changes are shared across different mental health problems.
• Symptom-specific neural alterations emerge in subcortical and cortical brain regions.
• Multi-modal MRI reveals distinct and shared brain trajectories from age 9 to 12.

## Graphical abstract

**Figure:**
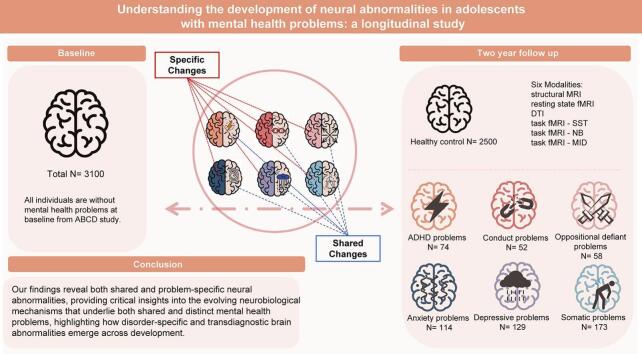

## 1 Introduction

Many mental health problems are neurodevelopmental in nature and have an onset during childhood or adolescence. Recent research suggests that approximately 22 % of the general population is impacted by a mental disorder, and nearly one in seven adolescents meet the criteria for a psychiatric diagnosis (Wittchen et al., 2011). The prevalence of mental health issues among adolescents has even started rising in recent years, which makes it even more urgent to understand how mental disorders develop.

A common hypothesis states that mental disorders may develop during adolescence due to the marked neurodevelopmental changes (Bethlehem et al., 2022). An increasing number of studies are employing longitudinal investigations to capture ongoing alterations and developments in the course of various disorders (Ai et al., 2020, Damatac et al., 2022, Oostermeijer et al., 2016). Indeed, studies have shown altered neurodevelopmental trajectories in individuals with ADHD (Soman et al., 2023a, Soman et al., 2023b) and depression (Yüksel et al., 2018). But those studies are conducted in participants that have already developed a disorder. It therefore remains unclear whether those neurodevelopmental differences reflect a preexistent vulnerability or a consequence of having developed mental health problems. In order to disentangle cause from consequence, longitudinal studies are required to assess participants in the prodromal phase. Meanwhile, existing trajectory studies are often restricted to a single disorder or modality (Romer et al., 2023), overlooking the shared symptoms and etiologies across mental disorders, and limiting the ability to capture complex brain interactions or provide a comprehensive understanding of their underlying neurobiological mechanisms. It therefore remains unknown whether differential neurodevelopmental trajectories could explain the vastly different clinical manifestations, or whether overlapping neuroanatomical changes are related to the development of mental health problems in general.

To investigate this, we selected participants from the Adolescent Brain Cognitive Development (ABCD) study (Jernigan et al., 2018), the largest longitudinal neuroimaging study focused in a critical developmental period from age 9–10. Our sample consisted of individuals who were initially healthy but later developed a range of mental health problems over a two-year follow-up period, and controls who remained healthy. Mental health problems were identified based on DSM-5 criteria and categorized into six distinct types: ADHD, anxiety, conduct, depressive, oppositional defiant, and somatic problems as assessed with the Child Behavior Checklist (CBCL 6–18) (Achenbach, 2013). We extracted structural and functional neuroimaging data collected at baseline and at two-year follow-up and investigated whether neural abnormalities already existed at baseline or developed together with the onset of mental health problems.

## 2 Method

### 2.1 Participants

The ABCD Study, which included 11,876 young people from age 9 to 10 at baseline, aims to understand the neurodevelopmental trajectories in adolescence and their relations to behavior, environment, and genetics. The study recruited participants from various schools and communities across the United States to gather a diverse cohort representing a broad spectrum of socio-economic, cultural, and geographic backgrounds. Participants undergo comprehensive assessments, which include neuropsychological tests, brain imaging, and various questionnaires about behavior, health, and lifestyle.

For our study, we selected individuals using the Child Behavior Checklist (CBCL), which provides critical information on participants' emotional and behavioral functioning (Jernigan et al., 2018). The CBCL is a widely used tool for assessing emotional and behavioral problems in children. The CBCL is designed to obtain a comprehensive picture of a child's functioning from the perspective of their parent or primary caregiver. The questionnaire covers various domains, including social interactions, academic performance, emotional regulation, and behavioral tendencies. The CBCL scores can be categorized into different syndromes, which provide valuable insights into potential clinical conditions or developmental issues. The use of standardized scores allows for comparisons across children and helps clinicians and researchers pinpoint specific areas of concern. We used CBCL DSM-oriented scales rather than KSADS-based diagnoses to define the groups of interest to limit drop-out because KSADS data were missing for up to 100 % of participants for several disorders at baseline, and to enable the comparison with prior ABCD based predictive studies.

**Fig. 1:**
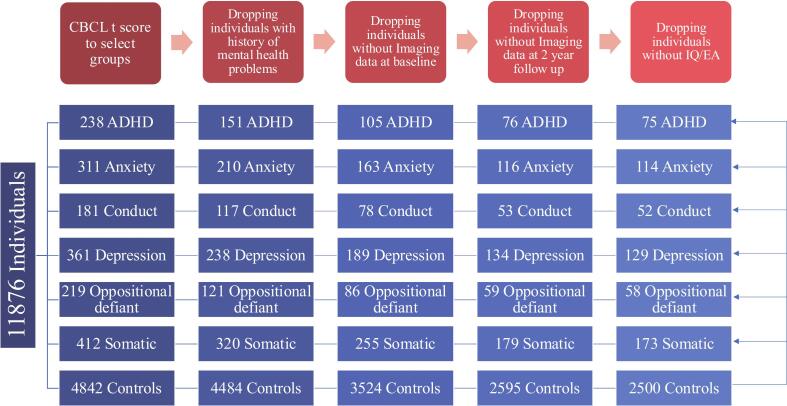
Flowchart illustrating the selection of research participants.

To investigate neurodevelopmental differences between children who developed clinically relevant mental health problems and those who remained healthy, we selected participants with a t-score < 65 on the DSM-oriented CBCL scales at baseline, and with t ≥ 65 at two-year follow-up. A cut-off of 65 provides the best balance in sensitivity and specificity in comparison with DSM classification based on structured clinical interview (Krol et al., 2006). By preselecting individuals with clinically relevant symptoms, the results will reflect altered brain development in children for whom clinical intervention would be appropriate(Patton et al., 2014). The results therefore do not reflect normative development which would be tested using regression in the complete sample. We identified 238 individuals with ADHD problems, 311 with anxiety problems, 181 with conduct disorder problems, 361 with depressive problems, 219 with oppositional defiant problems, 412 with somatic problems. In addition, we identified 4842 controls without clinically relevant symptoms (MCRS) (hereafter referred to as controls) with a t-score < 65 at baseline and at one and two year follow-up. After excluding participants with a psychiatric history at baseline (defined as having ever received mental health or substance abuse services) or without usable MRI data at baseline and/or follow-up (as large missing MRI datasets cannot be imputed), our final sample consisted of 74 individuals with first onset ADHD, 114 with anxiety problem, 52 with conduct problem, 129 with depressive problem, 58 with oppositional defiant problem, 173 with somatic problem, and 2500 controls (Table1**,** Fig. 1**.**). To reduce diagnostic overlap, individuals meeting criteria for both internalizing and externalizing groups at follow-up were excluded.

**Table 1:** Demographic data of the seven adolescents’ groups at baseline. The six disorder groups included participants without clinically relevant symptoms at baseline (CBCL t-score < 65) but with clinically relevant symptoms at two year follow-up (CBCL t-score ≥ 65). Controls did not have clinically relevant symptoms at baseline nor at follow-up (CBCL t-score < 65). P values are false discovery rate corrected.

	Control	ADHD	P	Anxiety	P	Conduct	P	Depress	P	Oppositional	P	Somatic	P
Sample size	2500	75		114		52		129		58		173	
Age (mean ± SD)^a^	9.93 ± 0.62	10.01 ± 0.62	0.39	9.83 ± 0.61	0.32	9.81 ± 0.62	0.2	9.81 ± 0.61	0.04*	9.82 ± 0.65	0.40	9.87 ± 0.62	0.20
Gender Male (%)^b^	48.36 %	61.33 %	0.07	50.88 %	0.67	48.08 %	1.0	37.98 %	0.04*	62.07 %	0.21	53.76 %	0.20
IQ (mean ± SD)^a^	103.62 ± 17.10	95.64 ± 14.86	7.09e-05*	102.07 ± 17.83	0.48	99.33 ± 14.54	0.08	102.12 ± 16.15	0.3	101.53 ± 15.88	0.43	100.30 ± 13.54	0.01*
EA (mean ± SD)^a^	17.01 ± 2.56	16.83 ± 2.21	0.48	16.69 ± 2.72	0.44	15.88 ± 2.76	0.02*	16.43 ± 2.86	0.04*	17.10 ± 2.48	0.79	16.46 ± 2.59	0.015*

a *t*-test.

b χ ^2^.

* Significant after FDR.

The ABCD Study was conducted under NIH/NIDA Protocol 2573, and approved by the Institutional Review Boards (IRBs), following the ethical guidelines set by the National Institutes of Health (NIH). Each site’s IRB provided oversight to ensure the protection of participant rights and compliance with federal regulations for research involving human subjects. The data that support the findings of this study are openly available in ABCD Study at https://nda.nih.gov/abcd, reference number 2573.

### 2.2 Imaging data

Imaging data included 1876 imaging features from six MRI modalities extracted from the ABCD dataset using the published protocol: structural MRI (sMRI), diffusion MRI (DTI), and functional MRI data (resting state (rsfMRI), task fMRI: the monetary incentive delay (MID), task fMRI: the stop signal task (SST), task fMRI: the emotional n-back (EN-back))(Hagler Jr et al., 2019). We selected 258 sMRI measures, 84 DTI measures, and 856 rsfMRI measures, 90 SST measures, 196 N-back measures, and 392 MID measures, including structural (volume, surface area, cortical thickness, fractional anisotropy) and functional (temporal variance, beta weights, and resting-state connectivity) brain measures across six imaging modalities, detailed in Supplementary Table S1. The ABCD image processing pipeline integrates both automated and manual methods, applying general and modality-specific corrections to account for issues such as head motion, image distortion, and intensity non-uniformity. A detailed description is available in Hagler Jr et al. (Hagler Jr et al., 2019).

### 2.3 Analysis

Statistical analyses were conducted in R to assess both baseline group differences and longitudinal neurodevelopmental changes associated with the onset of mental health problems. For each imaging feature, we estimated two separate linear regression models. To examine baseline differences, we tested the group effect for each feature at baseline $F_{0,i}$, correcting for age, sex, IQ, parental educational attainment (PEA), and scanner types (ST) at baseline (Senn, 2006).

$$F_{0,i}\;{\mathrm{Group}}_{i}+Age_{i}+Sex_{i}+IQ_{i}+PEA_{i}+ST_{0,i}$$

Harmonization procedures such as ComBat were not applied, as the clinical groups were distributed across many sites with small per-site sample sizes (typically fewer than 4 participants per group), which limits the stability and appropriateness of site-specific parameter estimation (Orlhac et al., 2022)

For the developmental model, we tested the group effect for each feature at 2 year follow-up $F_{2,i}$, correcting for baseline $F_{0,i}$, age, sex, IQ, parental educational attainment (PEA), and scanner types (ST) at both baseline and follow-up. This method controls for initial differences in brain values and yields unbiased estimates of group-related developmental differences:

$$F_{2,i}\;{\mathrm{Group}}_{i}+F_{0,i}+Age_{i}+Sex_{i}+IQ_{i}+PEA_{i}+ST_{0,i}+ST_{2,i}$$

Scanner model was included as a covariate at both timepoints to account for variance due to hardware differences and effectively also controls for study site effects, given that scanner and site are tightly coupled in the ABCD dataset.

Statistical tests were corrected for multiple comparisons in two steps. First, false discovery rate (FDR) correction (p < 0.05) was applied across all features within each imaging modality. Second, Bonferroni correction was applied across modalities (p < 0.05/6) when testing multiple modalities within a disorder, and across disorders (p < 0.05/6) when testing multiple disorders within a modality. In addition, to assess the degree of shared neurodevelopmental alterations between mental health problems, we computed spatial correlations between brain-wide t-value maps for each modality, which were similarly Bonferroni corrected across six modalities.

To assess individual-level neurodevelopmental trajectories, we additionally visualized longitudinal changes in each brain region that showed significant group effects in the above models. Specifically, we generated spaghetti plots to illustrate within-subject changes from baseline to follow-up, and violin plots to depict the distribution of change scores (follow-up minus baseline), residualized for age, sex, IQ, parental education, and scanner model. These visualizations were created in R using the ggplot2 package and provide a complementary view of inter-individual variability and within-group overlap in brain development.

To assess whether the univariate group-level results also translated to sufficiently large multivariate group differences that would allow identification of group membership at the individual level, we conducted a machine learning analysis on residualized delta features (follow-up minus baseline values after regressing out covariates (age, sex, IQ, parental education, and scanner model)). For each disorder × modality pair, we reduced the imaging features to a lower-dimensional representation using PCA (retaining 80 % of total variance), and then applied random forest classifiers to distinguish cases from controls. To address the substantial imbalance between patient and control groups, we randomly subsampled controls to match the number of cases in each repetition. Classification performance was evaluated using 10-fold stratified cross-validation, repeated 10 times with different subsamples. Model performance was summarized using mean and standard deviation of the area under the receiver operating characteristic curve (AUC) across repetitions. These analyses were conducted in Python using scikit-learn and provide an independent validation of the separability between groups under dimensionality reduction and resampling conditions.

## 3 Results

We compared six groups that consisted of participants who developed different mental health problems (ADHD, anxiety, conduct, depressive, oppositional defiance, and somatic problems; N = 58 to N = 173) with controls (N = 2500) without clinically relevant symptoms. There were several significant differences in demographic data between groups. To account for differences in demographic composition, all analyses were corrected for age, sex, IQ, parental EA and scanner model. In addition, we performed sensitivity analyses in which we matched the control group on these characteristics, for which the results remained comparable (see Supplementary Fig. S7-S10, Supplementary Table S6).

### 3.1 Baseline

**Fig. 2:**
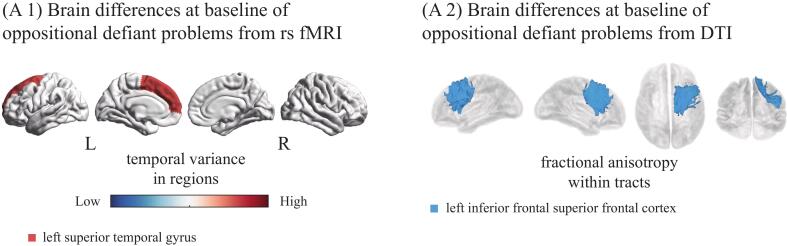
Aberrant brain regions in oppositional defiant problems at baseline. (A1) Oppositional defiant: increases in cortical rsfMRI variance, (A2) Oppositional defiant: decreased fractional anisotropy within DTI atlas tracts. Abbreviations: rs fMRI = resting-state functional magnetic resonance imaging; DTI = diffusion tensor imaging; L = left hemisphere; R = right hemisphere.

After false discovery rate (FDR) correction for multiple comparisons, we observed limited significant differences at baseline (before the onset of clinically relevant symptoms) for the conduct and oppositional defiant problem groups compared to controls in rsfMRI measures (Fig. 2**.**, Supplement Fig. S1), but not for ADHD, anxiety, depressive and somatic problem groups. The oppositional defiant problem group showed increased temporal variance in the left superior temporal gyrus at rest, and decreased average fractional anisotropy within tracts of the left inferior frontal superior frontal cortex and fibers included in the left hemisphere DTI atlas (Supplement Table S2). The conduct and oppositional defiant groups also initially showed increased temporal variance in the left superior frontal gyrus at rest. However, inspection of violin plots suggested that these effects were driven by extreme outliers (Supplementary Fig. S2-S3), and these effects did not remain significant after excluding those individuals from the analysis (Supplementary Table S2). We found no significant differences at baseline for any of the other neuroimaging measures. Thus, only individuals that later developed oppositional defiant symptoms already had prodromal regionally specific differences in rsfMRI measures.

### 3.2 Developmental trajectories

**Fig. 3:**
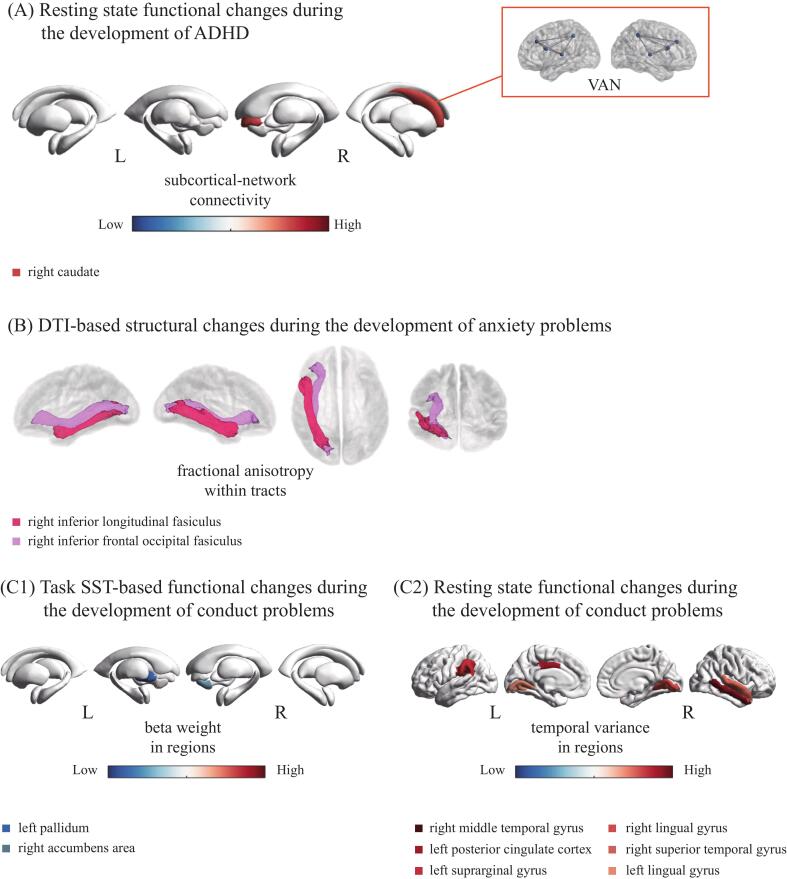
Aberrant neurodevelopment in ADHD, anxiety, and conduct. (A) ADHD: increases in subcortical functional connectivity; (B) Anxiety: average fractional anisotropy within DTI atlas tracts; (C1) Conduct: decreases in subcortical regions during performance of the SST, (C2) increases in cortical rsfMRI variance. Abbreviations: ADHD = attention-deficit/hyperactivity disorder; DTI = diffusion tensor imaging; rs fMRI = resting-state functional magnetic resonance imaging; SST = stop signal task; VAN = ventral attention network; L = left hemisphere; R = right hemisphere.

We next tested whether there were significant differences in neurodevelopment between the different symptom groups and controls. The results showed differential neurodevelopmental changes at two-year follow-up (after the onset of clinically relevant symptoms) for ADHD, anxiety, depressive, conduct, and oppositional defiant problem groups (Fig. 3**.**; Supplement Fig. S4), but not for the somatic problem group. Inspection of the violin plots showed extreme outliers for the depressive and oppositional defiant problem group analyses (and for one of thirteen variabels for conduct problems), and these results did not remain significant after excluding those individuals from the analysis (Supplementary Fig. S5; Supplementary Table S3). For ADHD problems, the results showed higher resting-state functional connectivity changes between the ventral attention network (VAN) and right caudate nucleus. For conduct problems, rsfMRI measures showed increased temporal variance changes in the right middle temporal gyrus, left posterior cingulate cortex, right lingual gyrus, left supramarginal gyrus, left lingual gyrus, right accumbens area and right superior temporal gyrus. In addition, this group showed decreased activity changes during the SST for correct stop versus incorrect stop in the left pallidum and right ventral diencephalon, and for correct stop versus correct go contrast in the left pallidum (see Supplementary Table S3). The anxiety problem group showed decreased diffusion changes in average fractional anisotropy within the right inferior longitudinal fasciculus and right inferior frontal occipital fasciculus. These results indicate that the ADHD, anxiety, and conduct problem groups had different neurodevelopmental changes after the onset of mental health problems, while no robust effects were found for the oppositional defiant, depressive and somatic problem groups.

### 3.3 Machine learning analysis

To evaluate whether neurodevelopmental changes were sufficiently large to identify participants on the individual level, we performed machine learning analyses on brain changes. Across all disorder × modality combinations, the average AUC values ranged from 0.45 to 0.52, with most estimates centered around 0.5, indicating limited separability between groups based on imaging features alone under these conditions (Supplementary Table S5).

### 3.4 Correlations

**Fig. 4:**
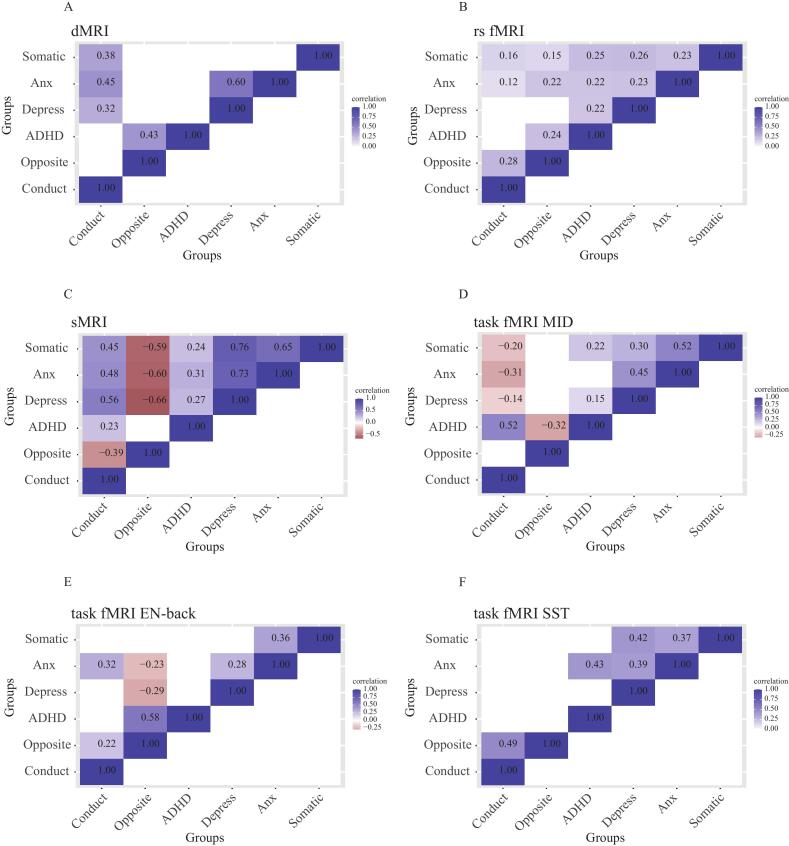
Correlations between different mental health problems in brain-wide abnormalities for different imaging modalities at baseline (blue is positive and red is negative). Only significant correlations between t-values from the baseline linear models at p(FDR) < 0.05/6 are shown. (A) DTI; (B) rsfMRI; (C) sMRI; (D) MID task fMRI; (E) EN-back task fMRI; (F) SST task fMRI. Abbreviations: ADHD = attention-deficit/hyperactivity disorder; DTI = diffusion tensor imaging; rs fMRI = resting-state functional magnetic resonance imaging; sfMRI = structural magnetic resonance imaging; SST = stop signal task; MID = Monetary Incentive Delay Task; EN-back = Emotional n-back Task; L = left hemisphere; R = right hemisphere. (For interpretation of the references to colour in this figure legend, the reader is referred to the web version of this article.)

To evaluate whether the neurodevelopmental changes were shared between symptom groups or specific per group, we performed pairwise brain-wide correlations for each MRI modality separately between the different mental health problem groups using t-values across the brain for the comparison with controls (Cohen et al., 2009). At baseline and after FDR correction for multiple comparisons, the results showed both comparable and opposing patterns of brain abnormalities before the onset of clinically relevant symptoms (Fig. 4**.**). While the ADHD, depressive, anxiety and somatic problem groups largely showed positively correlated brain differences compared to controls, the conduct and oppositional defiant problem groups showed negatively correlated brain differences with the other groups. Specifically, the oppositional defiant problem group showed negative correlations with several other groups in sMRI and task-related activity during the MID and emotional N-back task, and the conduct problem group showed negative correlations with other groups during the MID. Interestingly, the oppositional defiant and conduct problem groups also showed a negative correlation for sMRI, indicating that structural brain abnormalities were specific to the oppositional defiant problem group.

**Fig. 5:**
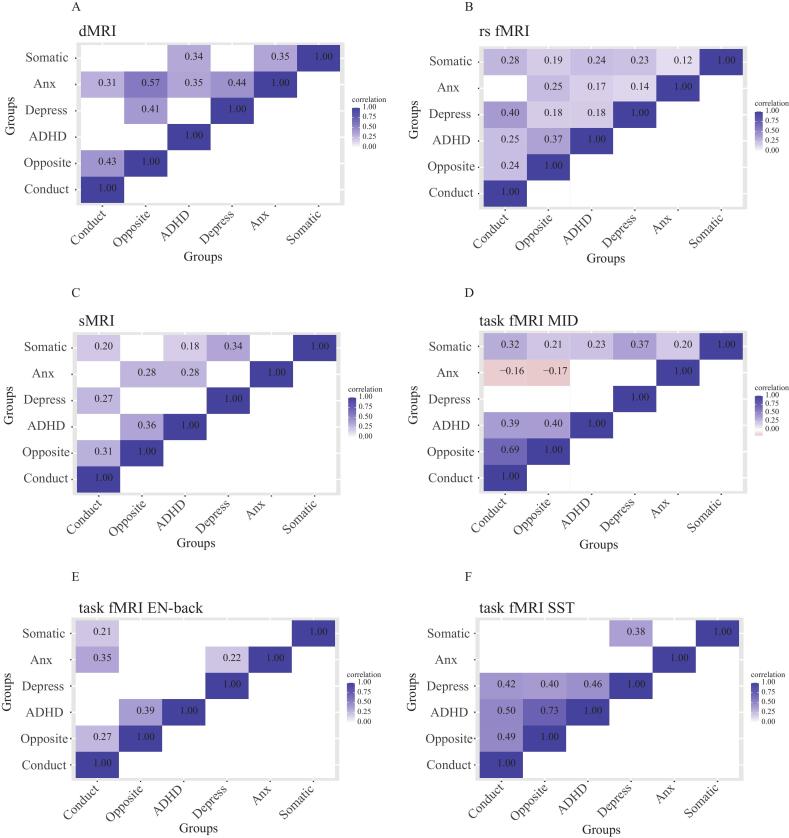
Correlations between different mental health problems in brain-wide abnormalities for different imaging modalities at follow-up (blue is positive and red is negative). Only significant correlations between t-values from the change linear models at p(FDR) < 0.05/6 are shown. (A) DTI; (B) rsfMRI; (C) sMRI; (D) MID task fMRI; (E) EN-back task fMRI; (F) SST task fMRI. Abbreviations: ADHD = attention-deficit/hyperactivity disorder; DTI = diffusion tensor imaging; rs fMRI = resting-state functional magnetic resonance imaging; sfMRI = structural magnetic resonance imaging; SST = stop signal task; MID = Monetary Incentive Delay Task; EN-back = Emotional n-back Task; L = left hemisphere; R = right hemisphere. (For interpretation of the references to colour in this figure legend, the reader is referred to the web version of this article.)

We performed the same correlation analysis for the changes in MRI modalities at two-year follow-up (after the onset of clinically relevant symptoms), which largely showed positive correlations for each of the group pairs and MRI modalities (Fig. 5**.**). The majority of correlations between groups were significant for rsfMRI, while approximately half the correlations were significant for structural MRI, DTI and task-based fMRI. No significant negative correlations were observed.

## 4 Discussion

This study investigated whether neural abnormalities already exist before the development of different mental health problems or whether those develop with the onset of mental health problems. Our results show that the onset of mental health problems in youth between 9 to 12 years of age is primarily associated with altered neurodevelopment during this critical period, with limited prodromal differences in brain structure and function. The longitudinal comparison to controls revealed focal neurodevelopmental changes that were specific for different mental health problems, while the brain-wide correlation analysis suggests that most of altered neurodevelopment was shared between symptom groups. Together, these results suggest that structural and functional brain abnormalities primarily emerge in parallel with the development of clinically relevant mental health problems, and that these contain both shared altered brain-wide neurodevelopmental trajectories as well as brain changes that are specific to particular symptoms.

Participants with ADHD, anxiety and conduct problems showed significant differences in neurodevelopment compared to controls. ADHD and conduct problems were mainly associated with changes in resting-state functional connectivity, while conduct problems also showed reduced task-based activity during behavioral inhibition. In the anxiety group, we observed additional developmental differences in white matter. While the changes in resting-state functional connectivity were largely comparable between groups, the results also showed specific changes. Problem-specific changes were limited to the cortex for conduct problems, and to cortical-subcortical interactions for ADHD problems. No significant differences were observed in individuals with depressive, oppositional defiant or somatic problems, which may only evolve after longer disease duration.

The neuroimaging results for the specific groups are in line with what has been previously reported for patients with longer disease durations. For those patients, it is unclear whether neural abnormalities were already present during the premorbid phase, related to the onset of symptoms, or a consequence of having a disorder (scarring). In this study, we identified few significant brain differences at baseline. We found increased temporal variance in the left superior temporal gyrus in the oppositional defiant problem group. Alterations in the left superior temporal gyrus have been observed in individuals with oppositional defiant problems (Noordermeer et al., 2016). Moreover, decreased fractional anisotropy in left inferior frontal superior frontal cortex and entire left hemisphere were found, which has also been reported in individuals with combined ADHD and oppositional defiant disorder (van Ewijk et al., 2016). Our results indicate that these cortical changes already occur before the onset of oppositional defiant symptoms, which may help identify early signs of oppositional defiant problems.

During the development of problems, we found higher functional connectivity between the VAN and the caudate nucleus in ADHD problem group. The VAN, which consists of regions within the ventral prefrontal cortex and the temporoparietal junction, can redirect attention toward salient stimuli, particularly novel stimuli (Walkup et al., 2021). Hypofunction of the VAN has previously been reported in ADHD (McCarthy et al., 2013). The caudate nucleus plays an important role in motor, emotional, and cognitive functions (Robinson et al., 2012), and the dysfunction of it has been associated with ADHD (Driscoll et al., 2020). Although no study has directly reported altered connectivity in adolescents with ADHD problems between the VAN and caudate nucleus, this altered connectivity may underlie the attentional and executive dysfunctions which are characteristic of ADHD.

During the development of conduct problems, we found increased temporal variance in six cortical regions. These regions have previously shown differences in brain structure and function in individuals with conduct problems (Fairchild et al., 2014, Hyatt et al., 2012, Jiang et al., 2015, Wu et al., 2017; J. Zhang et al., 2019, Zhou et al., 2015). In addition, we observed decreased activity in the pallidum in this group during behavioral inhibition. The pallidum is involved in the regulation of voluntary movement, inhibitory control and various cognitive and emotional functions (Gillies et al., 2017, Jones et al., 2020; S. Zhang et al., 2015) and decreased pallidum activity during behavioral inhibition might be related to the impulse control impairment associated with conduct problems.

In the anxiety problems group, we found developmental changes in white matter. Previous studies have reported decreased fractional anisotropy in the right inferior longitudinal fasciculus and right inferior fronto-occipital fasciculus in individuals with high anxiety and social anxiety disorder (Lu et al., 2018, Tükel et al., 2017). Our results indicate that the above neural abnormalities develop during the onset of clinically relevant symptoms, rather than that those reflect premorbid abnormalities or scarring effects.

The visualizations of subject-specific trajectories using spaghetti plots and violin plots revealed that the majority of group-level differences in brain development were not driven by outliers but instead reflected consistent shifts across many participants within each group. This is particularly important given the known heterogeneity of psychiatric trajectories during adolescence. By visualizing individual changes from baseline to follow-up, the results showed that several neurodevelopmental effects emerged gradually rather than being present at baseline. These findings strengthen the interpretation that the observed brain alterations reflect developmental changes associated with symptom emergence, rather than pre-existing group differences. However, the same visual inspections showed that some results were due to extreme values. We suspect that these extreme values reflect technological problems with image acquisition and/or processing, rather than true biological changes. As we do not have the original data to check the quality of the data, we chose to remove those participants from the analysis, which revealed that some of the results were generated by single neuroimaging scans.

The low to moderate AUC values observed across disorder × modality combinations suggest that, while our group-level models detected statistically significant effects, these effects do not readily translate into individual-level prediction. This discrepancy highlights that group-level statistical differences may capture average tendencies driven by subsets of individuals, while substantial overlap and heterogeneity between patients and controls limits discriminative power at the individual level.

While the longitudinal comparison to controls shows that multiple neural abnormalities are specific to particular mental health problems, the correlation analysis between symptom groups primarily showed positive correlations for all MRI modalities. This suggests that the different symptom groups also share an abnormal brain-wide neurodevelopmental trajectory. This concept fits well with the general structure of mental health problems, for which the most parsimonious model contains a general psychopathology factor with additional specific symptom clusters (Lahey et al., 2012). Together, our findings imply that altered brain development consists of a largely shared neurodevelopmental factor with additional symptom-specific brain changes.

While the comparisons between symptom groups and controls without clinically relevant symptoms hardly showed significant differences at baseline (before the onset of clinically relevant symptoms), the correlations between symptom groups at baseline did reveal abundant whole-brain correlation patterns of neural aberrancies. We found high correlations for structural (0.60–0.76) and moderate correlations for functional (0.23–0.52) abnormalities between groups with internalizing problems (depressive, anxiety and somatic problems) (Babicka-Wirkus et al., 2023), which were considerably stronger than for the positive correlations after the onset of clinically relevant problems (0.12–0.44). This may suggest that there is considerable overlap in neuroanatomy between internalizing groups in the prodromal phase. But as these problems developed in different directions, the correlations between the neuroanatomical differences weaken. Strikingly, even negative correlations were observed for the two groups with externalizing problems. The MID task measures of the conduct problem group were negatively correlated with those for internalizing problems. And brain activity measures during emotional working memory and structural MRI for the oppositional defiant group showed negative correlations with those for internalizing problems. This suggests that functional and structural brain abnormalities of the (externalizing) conduct and oppositional defiant groups already existed at baseline. Meanwhile, the findings for the internalizing groups reflect the comorbid nature of mental health disorders, as previous studies have shown that depression, anxiety problems and somatic problems often co-occur, sometimes with ADHD (Bekhuis et al., 2015, Campo, 2012, Lallukka et al., 2019, Reale et al., 2017, Riglin et al., 2021).

The current study is based on the large ABCD cohort study, which provides sufficient imaging data at baseline and follow-up for multiple mental health problems. Nevertheless, there are several limitations to this study. First, this study is based on children from North America, and whether the results also apply to children from other continents requires further investigation. Second, the children in this study were 9 to 10 years old at baseline, and we do not yet know whether the results also apply to children of younger or older age. Third, we included individuals who also had other problems as the resulting sample was not sufficiently large to exclude participants with comorbid problems. While this is beneficial for the generalization of the results to the clinical population, comorbid symptoms may have limited the identification of differences between problem groups. Fourth, we could not include family history of psychiatric disorders as a covariate due to its substantial missingness in the ABCD dataset at the time of analysis. Future studies incorporating more complete familial and environmental risk data will be important to provide complementary insights into neurodevelopmental trajectories. Lastly, our group definitions were based on symptom thresholds from the CBCL rather than formal diagnostic interviews, meaning that participants may represent high-risk or subclinical populations rather than individuals with confirmed psychiatric disorders.

In conclusion, our longitudinal study reveals complex neurodevelopmental changes in adolescents who develop clinically relevant mental health problems between 9 to 12 years of age. They have premorbid neural abnormalities that are different for internalizing and externalizing problems, have altered brain-wide neurodevelopmental trajectories that are in common, and additional symptom specific brain changes. These results contribute to our understanding how neural abnormalities evolve and relate to the development of distinct mental health problems.

## Declaration of generative AI and AI-assisted technologies in the writing process

During the preparation of this work, the authors used ChatGPT (OpenAI) to improve the clarity and expression of the manuscript text. After using this tool, the authors reviewed and edited the content as needed and take full responsibility for the content of the published article.

## CRediT authorship contribution statement

**Jiangyun Hou:** Writing – original draft, Visualization, Software, Resources, Methodology, Formal analysis, Conceptualization. **Laurens van de Mortel:** . **Weijian Liu:** Visualization. **Shu Liu:** Visualization. **Arne Popma:** Writing – review & editing, Supervision. **Dirk J.A. Smit:** Writing – review & editing, Supervision, Software, Methodology. **Guido van Wingen:** .

## Declaration of competing interest

The authors declare the following financial interests/personal relationships which may be considered as potential competing interests: Dr. Guido van wingen has received research support from Biogen, Bitbrain, Philips and GH Research for projects unrelated to this work. Msc. Jiangyun Hou, Dr. Laurens van de Mortel, Dr. Weijian Liu, Dr. Shu Liu, Dr. Arne Popma and Dr. Dirk J.A. Smit reported no biomedical financial interests or potential conflicts of interest.

## Data Availability

Data will be made available on request.
